# Effects of Genipin Crosslinking of Porcine Perilimbal Sclera on Mechanical Properties and Intraocular Pressure

**DOI:** 10.3390/bioengineering11100996

**Published:** 2024-10-02

**Authors:** John Riesterer, Alexus Warchock, Erik Krawczyk, Linyu Ni, Wonsuk Kim, Sayoko E. Moroi, Guan Xu, Alan Argento

**Affiliations:** 1 Department of Mechanical Engineering, University of Michigan-Dearborn, 4901 Evergreen Road, Dearborn, MI 48128, USA; riestere@umich.edu (J.R.); awarchoc@umich.edu (A.W.); ekrawczy@umich.edu (E.K.); wskim@umich.edu (W.K.); 2 Department of Biomedical Engineering, University of Michigan, Ann Arbor, MI 48109, USA; nilinyu@umich.edu (L.N.); guanx@med.umich.edu (G.X.); 3 Department of Ophthalmology and Visual Sciences, Havener Eye Institute, The Ohio State University Wexner Medical Center, Columbus, OH 43210, USA; sayoko.moroi@osumc.edu; 4 Department of Ophthalmology and Visual Sciences, Kellogg Eye Center, University of Michigan, Ann Abor, MI 48105, USA

**Keywords:** perilimbal sclera, genipin, crosslinking, fluorescence, perfusion, intraocular pressure, viscoelasticity, strain rate, tangent modulus, stress relaxation

## Abstract

The mechanical properties of sclera play an important role in ocular functions, protection, and disease. Modulating the sclera’s properties by exogenous crosslinking offers a way to expand the tissue’s range of properties for study of the possible influences on the eye’s behavior and diseases such as glaucoma and myopia. The focus of this work was to evaluate the effects of genipin crosslinking targeting the porcine perilimbal sclera (PLS) since the stiffness of this tissue was previously found in a number of studies to influence the eye’s intraocular pressure (IOP). The work includes experiments on tensile test specimens and whole globes. The specimen tests showed decreased strain-rate dependence and increased relaxation stress due to the cross-linker. Whole globe perfusion experiments demonstrated that eyes treated with genipin in the perilimbal region had increased IOPs compared to the control globes. Migration of the cross-linker from the target tissue to other tissues is a confounding factor in whole globe, biomechanical measurements, with crosslinking. A novel quantitative genipin assay of the trabecular meshwork (TM) was developed to exclude globes where the TM was inadvertently crosslinked. The perfusion study, therefore, suggests that elevated stiffness of the PLS can significantly increase IOP apart from effects of the TM in the porcine eye. These results demonstrate the importance of PLS biomechanics in aqueous outflow regulation and support additional investigations into the distal outflow pathways as a key source of outflow resistance.

## 1. Introduction

The sclera makes up most of the external shell of the eye [1] and is the eye’s primary load-bearing structure [2]. Its mechanical response is consistent with many other biological soft tissues, exhibiting characteristics of viscoelasticity and a response that initially stiffens with an increase in strain. The sclera’s mechanical properties are known to influence diseases such as glaucoma and myopia, as well as ocular functions, such as outflow [3] and injury prevention [4,5]. The perilimbal sclera (PLS) was shown to play a role in IOP regulation. In [3], the steady-state IOP of porcine eyes during whole globe perfusion showed a strong correlation with the tangent moduli of the PLS of the same eyes, but no significant correlations with the tangent moduli of the cornea or posterior sclera. Studies by others showed concentrated strain in the PLS with increasing IOP [6] and that the characteristics of the PLS are highly correlated with the IOP [7].

The nano- and microscale morphology of collagen was shown to have an effect on mechanical properties in individual collagen fibers [8,9], collagen matrices [10,11,12], sclera [13], and bone [14]. This morphology arises from a hierarchical structure in which tropocollagen is the smallest fiber-like unit. Tropocollagen naturally forms crosslinks with adjacent tropocollagen through enzymatic oxidation of the amine side chains near terminal ends as part of a dynamic mechanism to develop and maintain tissue properties [15,16]. Crosslinking also occurs non-enzymatically through glycation commonly associated with aging [17,18,19]. The extent of collagen crosslinking in the sclera, in contrast to collagen content, was shown to be a primary factor in mediating the tissue’s loading response [20].

Exogenously crosslinking [21] eye tissue is currently researched as a method to treat some structural diseases of the eye and also as a technique to stiffen the tissue to study its mechanical role in the eye’s behavior and relation to diseases such as glaucoma [22,23], keratoconus [24] and myopia [22,25,26]. Crosslinking can be induced by chemical, physical, and enzymatic agents [27]. Genipin is one of the few non-native crosslinking agents that forms long linkages between crosslinks due to its ability to polymerize with adjacent genipin molecules [27,28,29]. Studies have suggested genipin crosslinking results in a disruption of the native micro- and nanoscale structure of collagen by altering axial periodicity, even to the extent of eliminating striation in the fibril [30,31,32].

Uniaxial testing is a commonly employed and effective method of determining the conventional stress–strain and viscoelastic behaviors of soft tissue [33], and is particularly well-suited for straightforward sample-to-sample comparison of the effects of crosslinking on tissue. Porcine corneal strip samples treated with genipin were found to have significantly increased tensile stress at 6% strain compared to control samples [30]. Similar tests were conducted using genipin and riboflavin on porcine sclera and found to increase its stress and tangent modulus [34]. Tests on pericardium [35] and aortic valve [36] tissues showed reduced stress relaxation due to genipin. Another study [37] found that the extent of crosslinking of porcine sclera induced by genipin had a prominent effect on stress–strain properties, where higher densities of crosslinking led to increased ultimate stress and modulus. Softening induced by cyclic loading of tree shrew scleral samples was reduced using a genipin crosslinking treatment [38]. Results on the effects of genipin on tensile relaxation stress of the sclera do not appear to be available.

Whole and partial globe experiments can also be used for characterization via strain measurements [4,22,39,40,41], steady-state (SS) IOP [3,42,43], and outflow facility [44,45]. Determining the effects of crosslinking on a behavior of a whole globe is complicated by the potential for diffusion of the crosslinker into an unintended tissue. Fluorescence detection [27,32,46,47,48,49,50,51] can be used to verify the presence or lack of genipin in a tissue.

In the present work, the effects of genipin crosslinking of the PLS on the tissue’s stress–strain and viscoelastic properties were determined by uniaxial testing. A unique feature of these tests was the determination of the crosslinker’s influence on the tissue’s natural strain-rate dependence. Whole globe perfusion experiments demonstrated a significant effect of the crosslinker on the eye’s SS IOPs and outflow facility. Since TM stiffness is known to affect IOP in human eyes [52], the whole globe perfusion studies included a fluorescence assay procedure to verify the lack of genipin in the TM and to confirm its presence in the PLS of the whole globes. This work offers new insights into the role of genipin-crosslinked PLS on outflow in whole globes with an intact, non-crosslinked TM, the tissue’s strain rate dependence and its viscoelastic behavior.

## 2. Materials and Methods

### 2.1. Tissue Preparation

Whole porcine eyes were sourced from two slaughterhouses: Milligan’s Northwest Meat Market, Jackson, Michigan, USA, and Scholl Slaughterhouse, Blissfield, Michigan, USA. Both have been inspected and approved by the United States Department of Agriculture, Food and Safety Inspection Services for humane handling of livestock. Tissues were handled based on Institutional Biosafety Committee Approval Number IBCA00001946_AR02. The eyes were supplied with intact surrounding fat and connective tissue, and stored in a container maintained at 4 °C for approximately 1 h. The eyes were then dissected of fat, muscle, and connective tissues. During dissection, the eyes were periodically moistened with ophthalmic balanced salt solution (BSS, Alcon Laboratories, Fort Worth, TX, USA). Once fully trimmed, the eyes were placed into large centrifuge tubes containing soft tissue wipes moistened with BSS and the tubes were stored at 4 °C until the crosslinking procedure.

A 0.5% genipin solution was made by dissolving 5 mg genipin powder (Adooq Biosciences, Irvine, CA, USA) in 1 mL BSS. A spongy tissue (Pilling Wecksorb Weck-Wipes, Teleflex Inc., Wayne, PA, USA) was trimmed into small half-annulus pieces, with inner and outer radii of approximately 6 mm and 9 mm, respectively. These were soaked in the 0.5% genipin/BSS solution or BSS, the latter pieces for control eyes. One eye from each animal was randomly selected, by coin-flip, to be crosslinked and the fellow eye served as the control. The eye to be crosslinked was patted dry with tissue and three genipin-soaked pieces of sponge placed on its PLS, with no overlap, so as to evenly cover the entire region. Six drops from a 20 G needle of 0.5% genipin/BSS solution were administered evenly around the sponge every 5 min, for 30 min. The control eye was simultaneously treated the same way using BSS in the place of genipin. The globes were then rinsed with BSS and placed back into specimen tubes with moistened tissues in a 4 °C fridge for 18 h. Experiments were performed about 24 h after the animal was slaughtered.

### 2.2. Mechanical Stress–Strain and Relaxation Tests

**Figure 1:**
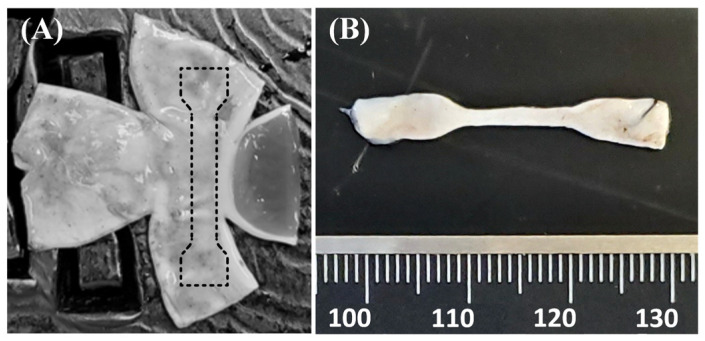
(**A**) A hemisphere of sclera dissected to lay flat so that a sample can be punched circumferentially from the perilimbal region, as shown by the sketch of the specimen superposed on the tissue specimen photo. (**B**) A punched, dog-bone-shaped sample. Scale is millimeters.

All mechanical tests were performed on a TestResources (Shakopee, MN, USA) tensile test machine (100Q225 system), fitted with a 9.8 N load cell. Samples were gripped in jaws patterned with small points on their gripping surfaces. These jaws are designed to effectively prevent specimen slippage. Each specimen was inspected post-test for signs of slipping in the wide, gripped region of the dog-bone-shaped samples. Characteristic longitudinal scrape marks caused by the jaw points during specimen slippage were not seen in any specimens. The test set-up includes a custom-made plexiglass chamber and lid that surrounds the specimen and the grips. During testing, 37 °C temperature was maintained in the chamber using a heating coil in a dish containing BSS for the duration of testing. Dog-bone-shaped samples were cut from the dissected hemispheres of control or genipin-treated globes using a custom-made TestResources cutting die. Samples were extracted from the PLS in the nasal and temporal regions, along the direction tangent to the cornea (Figure 1). Sample thickness was measured in three places in the gauge section using digital calipers. The samples were then placed in weighboats with a small amount of BSS and kept in the plexiglass chamber for approximately 15 min at which time they reached the porcine physiological temperature.

Uniaxial stress–strain tests were performed on the dog-bone samples at 3 speeds: 100 mm/min, 10 mm/min, and 0.5 mm/min (corresponding to strain rates of 0.128/s, 0.0128/s, and 6.4 × 10^−4^/s, respectively). The highest strain rate is indicative of a mild insult. The intermediate strain rate is used as a low-moderate rate to extract stress–strain data in line with similar research [53,54]. The lowest rate input approximates slower rates of pressure change that may occur in a globe, such as when moving from standing or seated positions to supine [55]. The percent strain rate dependence of the tissue stress is defined as $\left(\sigma_{2}-\sigma_{1}\right)/\sigma_{1}\times 100$, where $\sigma_{1}$ and $\sigma_{2}$ are the stresses resulting from tests at lower and high rates, respectively. (Modulus rate dependence is analogously defined). Collected stress–strain data were trimmed to remove slack-strain from the start of the toe region of each data curve. From the resulting curve, the mechanical effects of crosslinking were gauged using two measures: (1) the stress value at 8% strain, and (2) the tangent modulus of the linear region of the stress–strain curve. The 8% strain was selected in the first measure because it was approximately a transition point from the initial non-linear toe region to the linear region and is typically used by other researchers [34,54] to study the effects of crosslinking on sclera. The second measure was obtained by a linear fit of the data in the linear region and is very similar to the tangent modulus at the 8% strain.

Stress relaxation tests consist of two test segments, the input segment and the relaxation segment. For the input segment, the controller was set to run at a prescribed displacement rate and to terminate at the load that induces the prescribed stress level of 1 MPa in the specimen. This stress level was in the linear region of the stress–strain curve. Input displacement rates were 100 mm/min and 0.5 mm/min. The high-rate input approximates a step input and is critical for generating complete relaxation spectra of collagenous tissues from which the native tissue’s characteristic ability to relax is typically assessed [56]. Since some tissue relaxation occurs during input segment in the low-rate input case, it is not used to establish the tissue’s characteristic relaxation behavior. The low-rate relaxation spectrum reflects the eye’s viscoelastic behavior in a natural state in response to nominal inputs as might occur in the globe. By characterizing the tensile and viscoelastic properties at these strain rates, this work seeks to approximate the range of material properties exhibited by sclera tissue in vivo. For the relaxation segment, the displacement rate was set to zero, causing the grips to maintain constant positions so that stress relaxation occurs in the specimen. Stress relaxation data curves were cropped to include only the 300 s relaxation portion of the response. Relaxation stresses of control and crosslinked samples were compared at 10 s, 150 s and 275 s.

### 2.3. Whole Globe Perfusions

The experimental perfusion setup and preconditioning method used here have been described previously [3]. The perfusion experiments were conducted with the eye in a custom-built, water-jacketed globe holder which warms the posterior portion of the eye without allowing water to contact the eye. This was contained in a closed chamber to maintain hydration of the eye. Tubing (Tygon ND-100-65, ADF0004, 5/32 inch outer diameter, 3/32 inch inner diameter, Saint-Gobain North America, Malvern, PA, USA), a pressure sensor (Argon DTXPlus, Argon Critical Care Systems Singapore Pte. Ltd., Singapore), and valves were assembled and loaded with BSS. The pressure sensor data were continuously recorded using a data acquisition system (PowerLab 8/35, ADInstruments, Bella Vista, Australia). A 21 ½ gauge, 1 inch long needle attached to the free end of the tubing was positioned through the cornea and into the posterior chamber of the eye with careful insertion to avoid damage to the iris and lens. In the experiment, eyes were preconditioned using BSS as follows. The globe’s IOP was gradually increased to 15 mmHg over a two-minute period and then held constant for two minutes. A valve between the globe and the syringe was then closed allowing the tissue to relax and the globe’s IOP to accordingly decrease. After three minutes of relaxation, the cycle was repeated until the relaxation phase profiles stabilized [3]. This gradual establishment of venous pressure opens the collapsed vessels and valves in distal aqueous outflow pathways [57,58] and was shown to be a valid process when conducted on whole globes at physiologic pressures [59]. All eyes achieved a stable relaxation phase on the third cycle.

After preconditioning, the syringe was transferred to a syringe pump (New Era NE-100, New Era Pump Systems, Inc., Farmingdale, NY, USA) and the globe perfused using BSS at constant flow rate of 3 μL/min until reaching SS IOP, and then at 6 μL/min until the higher flowrate SS IOP was reached. Criteria for steady state required the average slope of the IOP across a ten-minute duration to be less than 0.001 mmHg/s. Net outflow facility, C, with units [(μL/min)/(mmHg)], was calculated using the standard definition given in Equation (1) [60], where $u_{1}$ and $u_{2}$ are, respectively, lower and higher perfusion flow rates with units [μL/min], and $P_{1}$ and $P_{2}$ are the corresponding measured IOPs with units [mmHg].

(1) $$C={(u}_{2}-u_{1})/\left(P_{2}-P_{1}\right).$$

### 2.4. Fluorescence Assay

Genipin crosslinked collagen will emit fluorophores at wavelengths above 630 nm when excited with wavelength of 590 nm [27,32,46,47,48,50,51]. An assay that takes advantage of this was used for qualitative [50] and quantitative [51] verification of crosslinking induced by genipin treatment in sclera and tendon, respectively. To verify that any measured differences in IOP in the perfused, crosslinked, globes were not due to inadvertent crosslinking of the TM, quantitative measurements of fluorescence were made of the TM from the whole globes used in the perfusion experiments. Fluorescence was also measured in the PLS of the same globes to confirm crosslinking in those tissues.

**Figure 2:**
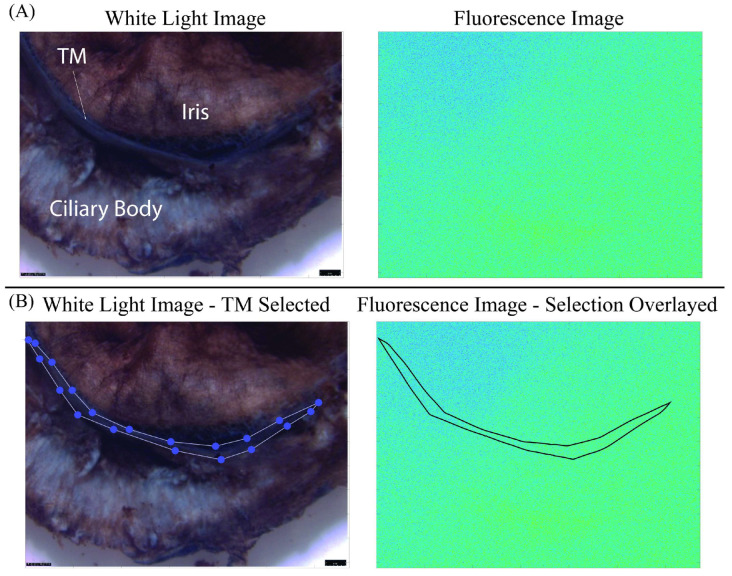
Process for evaluating TM fluorescence intensity values. (**A**) White light and fluorescence images of a tissue segment showing iris, TM, and ciliary body. (**B**) White light image of the manually selected section of the TM, along with its mapped location on the fluorescence image.

TM and samples of PLS were extracted from eyes immediately after the perfusion experiments. Samples of sclera, used for crosslinking verification, were extracted from around the PLS using a 10 mm diameter biopsy punch. The TM was removed from the whole globes by first resecting the anterior segment of the eye, then peeling the TM, while attached to the iris, from the underlying sclera. Figure 2A shows the anterior view of a circumferential segment of the excised TM attached to iris and ciliary body.

Images of all samples were acquired using an epifluorescent microscope (Leica M205 FA, Leica Microsystems, Deerfield, IL, USA) with a CY5 filter with 590–650 nm excitation and 662–737 nm emission. White light and fluorescence images were captured of portions of TM (Figure 2A). A custom MATLAB (The MathWorks, Inc., Natick, MA, USA) script was used to map the TM location from the white light image to the fluorescence image (Figure 2B).

A criterion for excluding the TM of treated, perfused globes was established based on the background autofluorescence of a non-treated group of TMs. Specifically, a quantitative assessment of the fluorescence intensity of all TMs extracted from the perfused globes was made by comparing their fluorescence intensities to two sets of reference data: a non-treated group and a treated group. Reference data were collected as follows. First, fresh eyes were obtained, and the TM was dissected using the previously described methodology. The non-treated group was imaged immediately after dissection, without receiving genipin treatment, to determine their autofluorescence. The treated group was then created by directly treating the TM of the same samples from the non-treated group with genipin. The treated group was imaged following genipin treatment.

The set of TMs from the perfused globes, along with both reference groups, underwent fluorescence imaging to capture emission within the range of 662–737 nm. The average fluorescence intensity, as measured by the mean pixel value for all pixels capturing the TM, in this fluorescence emission range was calculated. To establish a criterion for excluding perfused samples, a distribution plot was generated of the average fluorescence intensities of the non-treated TM reference samples. The upper limit of the non-treated distribution plot (235 A.U., see results) was set as the threshold to exclude perfused, treated, globes whose TM fluoresced. This limit of fluorescence intensity ensures the TM of every included eye had fluorescence intensity within the background fluorescence range including every TM sample in the non-treated reference group. Thus, any TM from the treated, perfused group with fluorescence greater than 235 was excluded.

### 2.5. Statistical Methods

Statistical and numerical calculations were conducted using MATLAB, Minitab (Minitab, LLC, State College, PA, USA), and Excel (Microsoft Corporation, Redmond, WA, USA). From each curve of the stress–strain tests the stress at 8% strain and the tangent modulus of the linear region were determined. In the stress relaxation tests, the relaxation modulus, which is the relaxation stress normalized by the constant input strain, was obtained from each curve. In both the stress–strain and relaxation tests, descriptive statistics (mean, standard deviation, and sample size) are reported for each mechanical quantity, and the coefficients of determination, R^2^, are also presented for the tangent moduli determined from linear regressions. In order to study the effects of crosslinking, the mechanical quantities of the genipin-treated samples were compared to those of the control samples, and paired, two-tailed, *t*-tests were performed at the 5% level of significance to evaluate the statistical significance of difference. To evaluate the strain rate dependence of stress–strain response, the stresses at 8% strain and the tangent moduli were compared at the three strain rates and corresponding *p*-values were determined from the unpaired, one-tailed, *t*-tests with equal variance. For the statistical comparison of the SS IOP of the genipin-treated samples to those of the control samples, unpaired, one-tailed, *t*-tests were performed. An unpaired, one-tailed, t-test with unequal variance was used to compare the fluorescence intensity means of excised TM samples from treated and perfused whole globes and reference treated TM samples.

## 3. Results

### 3.1. Mechanical Tests

**Figure 3:**
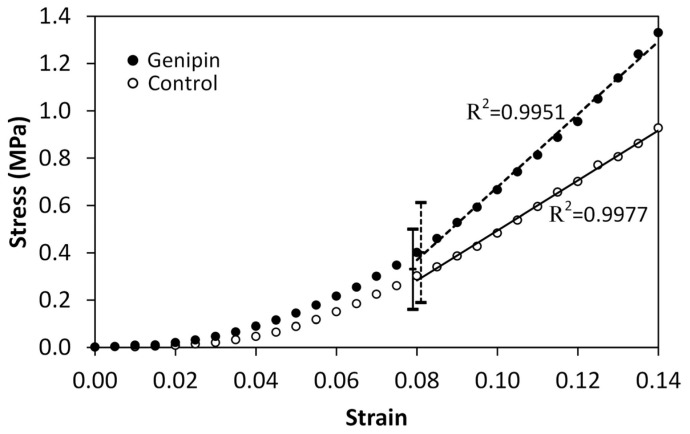
Representative control and genipin crosslinked stress–strain curves at the strain rate of 0.0128/s. The slope lines shown are the tangent moduli of the linear portions of the curves (R^2^ = 0.9977 control and R^2^ = 0.9951 crosslinked). The vertical solid and dashed lines at 8% strain are the stress ranges (mean ± standard deviation) of control and genipin crosslinked samples, respectively.

Representative stress–strain curves are given in Figure 3 and the results for the tangent modulus, stress at 8% strain and strain rate dependence are summarized in Table 1. Figure 3 shows a highly correlated tangent modulus from the curves’ linear regions to a linear model for both the control and the treated cases. This was typical for all the trials. The fifth column of Table 1 shows that the tangent moduli of the genipin-treated samples were significantly greater than those of the control samples for the two lowest strain rates (6.4 × 10^−4^/s and 0.0128/s). There was no significant difference between the control and treated tangent moduli at the highest strain rate (0.128/s). In the fourth column, at the lowest strain rate, the stress at 8% strain of the genipin-treated samples is seen to be significantly higher than that of the untreated control, but this effect did not occur at either of the higher tested strain rates. In Figure 3, the vertical solid and dashed lines at 8% strain show the stress ranges (that is, mean ± standard deviation presented in Table 1) of control and genipin crosslinked samples, respectively, at the intermediate strain rate of 0.0128/s. It is seen that the mean stress of the genipin-treated samples is higher than that of the control, but in this case, the difference is not statistically significant, as indicated in the table.

The sixth and seventh columns of Table 1 show the percent increase of each measured stress and tangent modulus at the two higher rates relative to the quantity’s value at the lowest rate. The corresponding statistical significance of the numerical increase of the measured quantity is also given. At the intermediate rate, only the increase in stress of the control is significant. At the highest rate, the values of all four quantities are significantly increased from their lowest rate values, but the rate dependence is smaller in the crosslinked case than in the control case. Specifically, the percent increase in the stress of the control case is 2.6 times that of the crosslinked case. Likewise, the percent increase in the modulus of the control case is 2.0 times that of the crosslinked case.

**Figure 4:**
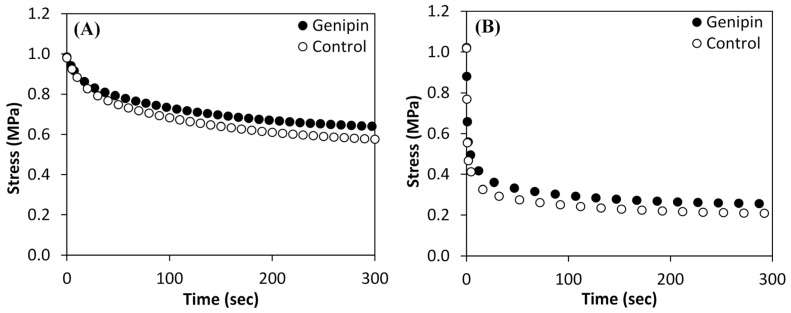
Representative stress relaxation curves of genipin crosslinked and control samples at input test speeds of (**A**) 0.5 mm/min (6.4 × 10^−4^/s) and (**B**) 100 mm/min (0.128/s).

The typical stress relaxation curves are shown in Figure 4. In the stress relaxation tests, the samples were stretched at 0.5 mm/min (Figure 4A) or 100 mm/min (Figure 4B) until the measured stress reached 1 MPa, then the resulting strain was held constant. The constant input strains of the genipin crosslinked and control samples were 0.17 and 0.19, respectively, when the test speed was 0.5 mm/min (Figure 4A). In the 100 mm/min test case (Figure 4B), the genipin and the control samples stretched to the same constant input strain, 0.14. For both input rates, the genipin crosslinked sample case relaxed less than the control sample case at all times, and to approach a greater equilibrium stress. Table 2 gives the results of the comparison of the measured stresses, normalized by input strains, of the control and crosslinked cases at three times during relaxation: 10, 150, and 275 s. The normalized relaxation stress of the genipin sample case is significantly greater than that of the control case for all three times and for both input strain rates.

### 3.2. Whole Globe Perfusions and Fluorescence Assays

**Figure 5:**
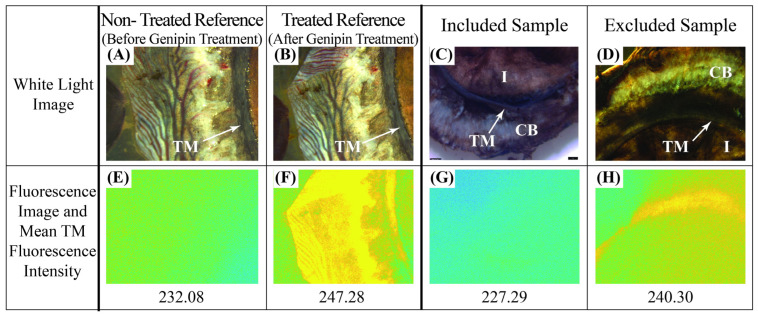
(**A**,**B**) White light images of a typical TM before and after genipin treatment, respectively, following extraction from one of the non-perfused globes. (**E**,**F**) Corresponding fluorescence images of (**A**,**B**) and the measured relative fluorescence intensities of the TM region of the image. This process was conducted on 4 TM samples to determine the intensities of the untreated and treated reference groups, as well as the intensity for rejecting treated, perfused globes. White light (**C**) and fluorescence (**G**) images of a TM extracted from a perfused, treated globe. This sample had fluorescence intensity (227.29) below the threshold intensity (235) established from the reference group and shown in Figure 6, and so its globe was included in the data set. White light (**D**) and fluorescence (**H**) images of a TM sample extracted from another perfused, treated globe. This sample had fluorescence intensity (240.30) above the threshold intensity, and so its globe was excluded from the data set. In (**C**,**D**), CB and I denote ciliary body and iris, respectively.

**Figure 6:**
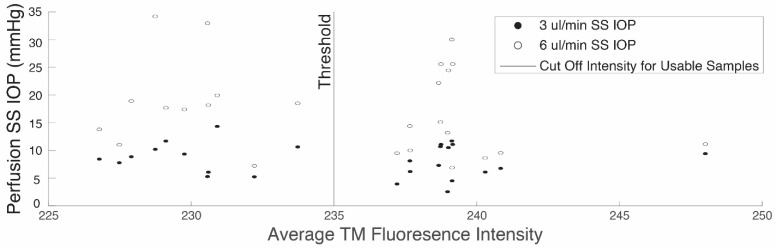
Representation of distribution of fluorescence intensity values. Scatter plot of fluorescence intensity of excised TM samples from genipin-treated, perfused globes. The vertical axis denotes the SS IOP of a perfused eye at the flow rates of 3 μL/min (black circle) and 6 μL/min (white circle). The threshold value for fluorescence intensity was chosen as the upper limit of the non-treated reference distribution.

Intensity measurements of the fluorescence of the tissues were determined using the fluorescence assay applied to images like those shown in Figure 5. The fluorescence intensities of TMs from the non-treated reference group were used to determine the threshold fluorescence intensity value for exclusion criteria. Figure 5E shows an example of a reference TM before treatment and Figure 5F shows the intensity of the same TM in Figure 5E after treatment. The threshold for rejection of a perfused, treated globe was chosen to be the upper limit of the intensity distribution of the non-treated reference group. These values ranged from 232 to 235. Thus, all perfused globes with TMs with fluorescence intensity measures greater than 235 were excluded from the data set. The fluorescence intensities of the non-treated reference TMs were found to be significantly lower than the treated reference TMs (*p* = 0.011). Figure 6 gives the scatter plot distribution of average fluorescence pixel intensities in the TMs from all the perfused, treated globes. The vertical line shows the exclusion criteria (235) taken from the nontreated reference TMs. Applying this threshold as an exclusion criterion led to the exclusion of 14 out of the initial 25 perfused, treated globes. These are the 14 pairs to the right of the vertical line at 235 in Figure 6. To confirm the validity of this exclusion criteria, we compared the fluorescence of the TMs of the included set of perfused globes to that of the treated reference TMs. The former was found to be significantly lower than the latter (*p* = 0.0012). Also, measurements of fluorescence intensities of the PLS samples punched from the treated, perfused globes indicated every PLS was successfully crosslinked.

**Figure 7:**
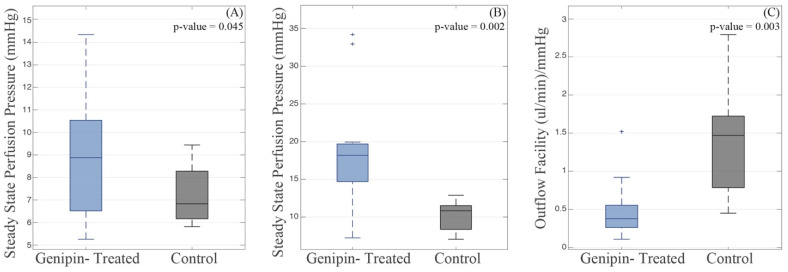
SS IOPs of whole porcine globes subjected to constant flow rates of (**A**) 3 μL/min and (**B**) 6 μL/min; (**C**) net outflow facility. *p*-values for one-sided, unpaired t-test are labeled for comparison between groups within each respective graph. Sample size is 11 and 9 for genipin-treated and control groups, respectively. Each data set includes a horizontal line representing the median, a box representing the 25th to 75th percentile, whiskers representing the data range (not including outliers), and outliers denoted by ‘+’.

Figure 7 summarizes the SS IOPs for low and high flow rates, along with the resulting net outflow facility, from the whole globe perfusion experiments. Statistical analysis revealed significant differences in the steady-state intraocular pressures between the control and the genipin-treated eyes. At a flow rate of 3 μL/min, the mean SS IOPs were 7.21 mmHg and 8.91 mmHg for the control and crosslinked globes, respectively, with standard deviations of 1.28 and 2.79 mmHg. This difference was significant with a *p*-value of 0.045. Similarly, at a flow rate of 6 μL/min, the mean steady-state IOPs were 10.07 mmHg and 19.08 mmHg for control and crosslinked globes, respectively, with standard deviations of 1.98 and 8.12 mmHg, respectively, and a *p*-value of 0.002. Additionally, the mean net outflow facility was determined to be 1.40 (µL/min)/(mmHg) for control eyes and 0.51 (µL/min)/(mmHg) for crosslinked eyes (*p*-value = 0.003, standard deviation of 0.73 and 0.41 for control and crosslinked, respectively). The resulting increases in the steady-state IOP due to crosslinking are 23.6% and 89.5% at 3 μL/min and 6 μL/min, respectively, while the outflow facility decreases by 63.6%.

## 4. Discussion

**Effects of crosslinking on IOP:** The focus on the perilimbal region for scleral crosslinking was partly motivated by previous research on non-crosslinked eyes that demonstrated a strong correlation between PLS stiffness (tangent modulus) and SS IOP [3], as well as other studies relating the behavior of the PLS to IOP [6,7]. The specimen tests, Table 1, showed a significant increase in the tangent modulus by crosslinking for testing strain rates of 0.0128/s or lower. To determine the effects of property modification induced by crosslinking on the outflow, the PLS of whole globes were genipin crosslinked for the perfusion experiments. The effect of the crosslinked PLS on the eye’s outflow was gauged via a comparison of the SS IOPs of globes with crosslinked and non-crosslinked PLS. Figure 7 indicated a significant increase in the SS IOPs of the crosslinked globes at both flow rates. SS IOP is a measure of outflow facility, which is the ratio of constant flow rate to the resulting SS IOP [60]. Thus, at the same perfusion flow rate, a lower SS IOP indicates a greater outflow facility than does a higher SS IOP. The net outflow facility, a measure based on both steady-state pressures, was also found to be significantly reduced by crosslinking.

A novel feature of the present work was strict quantitative control to ensure that eyes with TM that were inadvertently genipin crosslinked were excluded from the perfusion study. Though it is thought that the TM offers the primary resistance to outflow, and its resistance is dependent on its stiffness [52,61,62,63,64], the present experiments, therefore, suggest that elevated stiffness of the PLS can significantly increase IOP apart from the effects of the TM in the porcine eye.

Crosslinking of soft tissue is known to reduce crimp in the collagen structure [65], which will tend to shorten the stress–strain toe and effectively stiffen the tissue. In a study on collagen morphology in relation to age and spatial patterns [66], it was found that compared to other regions of the eye, the PLS exhibited the largest reduction in crimp due to aging, making collagen within this region unique in the sclera. The effect of crosslinking the posterior of whole globes was investigated in [67] using IOP and strains estimated from image tracking data. The study found that the slope of the toe region, in a graph of IOP vs. strain, was significantly greater in some crosslinked groups than in the paired control globes. The present results, which show a significant increase in IOP in eyes with crosslinked PLS, illustrate the potential importance of the PLS to the eye’s biomechanics pointed out in [66].

The effect of crosslinking of posterior sclera on outflow was not part of the present study. This is felt to be a reasonable limitation given previous results showing the strong correlation of native (i.e., non-crosslinked) PLS stiffness to IOP and the weak correlation of stiffness of the posterior sclera [3]. Though it is possible that the effects of exogenous crosslinking on the collagen microstructure might cause a pronounced influence on the posterior sclera that did not appear in the study on native stiffness [3], genipin crosslinking of posterior sclera in Norway rat eyes was found to have no effect on IOP [68].

**Effects of crosslinking on strain rate dependence:** Strain rate dependence refers to the increases in stress and modulus, and the decrease in failure strain observed in most materials, including ocular tissues [55,69,70], when loaded at higher rates relative to quasi-static rates. The testing rates chosen in this study span a moderate range commonly experienced by the eye. The lowest test rate, 6.4 × 10^−4^/s, represents a quasi-static input. The intermediate rate, 0.0128/s, is a low-moderate rate input in the range of the rate (0.07/s) between that of eye rubbing and non-contact tonometry [55]. Increases in stress and moduli were studied in human sclera [70] at tests up to 0.033/s and in bovine eye tissues [69] at tests up to 50/s. The highest strain rate used in this work, 0.128/s, was chosen to study the effect of the crosslinker during moderate-rate events, and also as a near-step input for the viscoelastic relaxation experiments.

As expected, and found in other quasi-static tests of crosslinked sclera [34,54], both the stress and modulus were significantly increased by genipin crosslinking at the lowest testing rate in the present work (Table 1). However, at the highest rate, although the stress at 8% strain and the modulus in the linear region were, respectively, increased by 6.0% and 9.6% on average by the crosslinker, the differences between the control and crosslinked cases were not statistically significant. This rate, 0.128/s, did induce statistically significant increased stress and modulus relative to the lowest rate of 6.4 × 10^−4^/s for both the control and crosslinked cases (Table 1, sixth and seventh columns). Crosslinking substantially reduced strain rate-dependent increase in stress and modulus relative to the controls (56.1% vs. 146.4% for stress and 23.7% vs. 48.4% for tangent modulus).

The reduction in the sclera’s strain rate sensitivity observed here could be related to how the collagen responds at low strain when it is genipin crosslinked. As stated above, exogenous crosslinking stiffens tissues and increases stress by causing the collagen fibers to uncrimp [65]. As natural crimp of the collagen fibers is understood to be responsible for the low modulus of the toe region [71,72], the straightening of the collagen fibers prior to mechanical loading by crosslinking may shorten the toe region by decreasing the strain values at which the collagen fibers become aligned and the tissue begins to exhibit a higher modulus. Related studies on decellularized collagenous arteries have found similar relations between short toe regions and straighter collagen alignment prior to loading [73]. Also, the results from a molecular dynamics study [74] suggest that crosslinking may inhibit uncoiling in tropocollagen molecules, affecting rate dependence. Therefore, it is possible that the diminished rate effects measured in the crosslinked samples are the result of pre-stiffening of the tissue due to the effect of the crosslinker on the collagen matrix, and this limits additional stiffening that normally occurs during higher rate loading. It is also possible that the high-rate stress and modulus might be significantly different from those of the low-rate case at higher strains since the deviation between the curves which begins in the toe increases with strain. Other published work showing diminished effects of a crosslinker on sclera at increased strain rates appears to be unavailable.

**Effects of crosslinking on viscoelasticity:** Viscoelasticity, including rate dependence, in biological tissues is necessary for their function. The increased modulus and stress produced by natural rate dependence in the eye serve to progressively reduce deformation during higher-rate events. This allows the eye to function normally over a wide range of strain rates. Analogous functions of viscoelasticity are present in other tissues, such as the spine, where rate dependence of spinal ligaments are protective at a high rate [75] by limiting deformation. The present results show that rate dependence is substantially lower in crosslinked sclera than in controls. The effects of this on the eye’s functionality during higher-rate events are unknown.

The viscoelastic tests were conducted at two input rates, a slow rate to reflect nominal inputs to the eye, and a high rate commonly used to characterize viscoelastic properties and to also study the eye’s response after a more severe insult. As the sclera deforms during normal biomechanical processes in the eye, it continuously dissipates energy. The low-rate input cases (Figure 4A) relaxed to a higher stress than the high-rate cases did (Figure 4B). This is because the viscoelastic tissue dissipated energy during the input phase, which occurred over a longer time in the low-rate input case. Thus, compared to the high-rate input case, there was less input energy remaining stored in the tissue at the end of the low-rate input to be dissipated during the relaxation phase.

Figure 4 and Table 2 show that after the input phase, the crosslinked cases relaxed to significantly higher stresses than the control, for both input rates, at 10, 150, and 275 s. Increased relaxation stress occurred due to the increase in the tissue’s stiffness by the crosslinking agent and demonstrates its effect on the tissues across the complete relaxation period. A study of bovine cornea also demonstrated that crosslinking can increase the relaxation stress for a particular strain [76]. The potential importance of the equilibrium stress to disease was studied in an investigation of early experimental glaucoma monkey eyes in which it was shown that the equilibrium stress of the early glaucomatous eyes was significantly greater than that of the non-glaucomatous eyes [77].

The high-rate input relaxation test is used to characterize viscoelastic materials because it minimizes energy dissipation during the input phase. Comparison of the control and genipin stresses at the same times in Figure 4B and Table 2, shows that the genipin-treated tissues subjected to a high-rate process relaxed more slowly than the untreated tissues. This also means that the treated tissues will dissipate energy more slowly than the controls during the relaxation period after a high-rate input. These results are consistent with another study [78], which demonstrates that individual collagen fibrils, when treated with an exogenous crosslinker that is able to form crosslinks between microfibrils, increase both the short and long relaxation times of the collagen. Although the low-rate input case relaxed less than the high-rate case, both had similar general relaxation behavior after crosslinking showing that the effect of the cross-linker on the tissue’s microstructure persisted despite the large loss of viscoelasticity that occurred during the slower input phase.

Though exploring the use of genipin as a scleral treatment was not the motivation of this work, the results showing increased IOP and relaxation time and decreased rate dependence in crosslinked PLS indicate the full range of changes in the eye’s natural behavior by scleral crosslinking are not yet entirely understood. Other work has indicated potential exacerbation of glaucoma due to crosslinked sclera [23] in a mouse model.

**Fluorescence assay to exclude crosslinked TMs:** The fluorescence assay used in this study prompted a criterion to exclude eyes from the whole globe perfusion study with inadvertent genipin crosslinking of the TM. Prior studies have used the fluorescence intensity of genipin-crosslinked collagen as a measure of the extent of crosslinking in tissues [51,79,80] or hydrogels [32,46,48,49,81]. In this work, a quantitative process to assess the genipin crosslinking intensity of TM was developed. A similar method was used on posterior globe tissues crosslinked with fluorescently labeled glutaraldehyde using image gain as a measure [79].

Native tissue can exhibit autofluorescence which contributes to low levels of background fluorescence in all captured fluorescence images [82,83]. The untreated reference TM fluorescence images (Figure 5E, for example), used in the reference group to create a threshold value for TM fluorescence, also served to provide a baseline of autofluorescence. This background autofluorescence, without quantification, could be incorrectly interpreted as genipin-induced fluorescence. Comparing reference-non-treated and reference-treated TMs indicated a significant difference between the fluorescence intensities of the groups (*p* = 0.011). Additionally, after applying the exclusion criteria to the perfused globes, the TM fluorescence intensity values of the remaining set of globes were also significantly lower than those of the treated reference TMs (*p* = 0.0012).

Background autofluorescence of native tissues poses a difficulty to interpreting fluorescence images as used in the assay. This is evident in Figure 5E, where a non-treated TM exhibited non-zero fluorescence. Contrary to other methods which allow for background autofluorescence quantification before treatment [84,85], the present study did not allow for dissection and fluorescence imaging prior to whole globe perfusion testing and thus obtaining an image to quantify background fluorescence prior to the genipin treatment was impossible. The method demonstrated here using reference sample groups allowed for conclusive judgements on the perfused samples’ TMs since an average background autofluorescence level was established.

Despite careful application of the crosslinker to a narrow ring of tissue surrounding the cornea on the exterior sclera, 14 of the 25 globes had TMs that exceeded the threshold set in this study for genipin crosslinking of the TM and so were excluded. This threshold was based on the background fluorescence levels of non-treated TMs, thus, every included globe had a TM that fluoresced within the range of background autofluorescence levels of the group of non-treated reference TMs.

**Preconditioning:** Preconditioning was not conducted in the uniaxial experiments. Although the repeated loadings and unloadings used for preconditioning can cause the specimen to reach a stable state, the specific mechanisms induced by the process to do so are not well understood [86] and could possibly result in remodeling the tissue’s microstructure [70]. Thus, there are merits to either approach and because of this uncertainty, some studies conduct analyses using both preconditioned and raw data [87,88] and indicate the effects of preconditioning may be tissue- and rate-dependent. In the present study, as in [54,89,90], for example, where each sample was handled and tested identically and compared only to each other, using non-preconditioned data is an acceptable approach in lieu of using both preconditioned and non-preconditioned data. In contrast to the uncertainty concerning preconditioning specimens in tensile tests, the decision to precondition whole globes is straightforward because the tissue in the globe is loaded in a physiologic manner during the process. The validity of preconditioning whole globes at physiologic pressures was recently confirmed by other researchers [59]. Thus, for the perfusion experiments, preconditioning was conducted on the whole globes at the onset of the tests.

## 5. Conclusions

Ocular aqueous humor outflow includes flow through vessels residing in the PLS. An open question is whether the interaction of the PLS with the vessels could impact IOP and thereby suggest the importance of the PLS properties on glaucoma. This work studied how changes to the properties of the PLS using genipin crosslinking affect IOP. Tensile tests of PLS specimens demonstrated the influence of a genipin treatment of porcine PLS on the mechanical stress–strain and viscoelastic relaxation properties of excised samples, including novel results showing that genipin increases the tissue’s relaxation stress, and reduces its strain rate dependence. In whole globe perfusions, eyes with genipin crosslinked PLS had significantly increased SS IOP and reduced net outflow facility. A quantitative genipin assay method was developed to exclude the TM as the driver of increased pressure. Identification of the changes to the PLS’s microstructure by the crosslinker and the resulting specific changes to its properties that were responsible for the measured increases in SS IOP were beyond the scope of this work. Such a study, along with the identification of the physical biomechanical mechanism at play, could form the subject of future work.

## Figures and Tables

**Table 1:** Results from stress–strain tests for control and genipin crosslinked, porcine, PLS samples. Comparisons of control and crosslinked stresses at 8% strain and control and crosslinked tangent moduli from linear regions of stress–strain curves. Superscripts on the *p*-values indicate the two cases compared. n = number of samples; stress and modulus values given as mean ± standard deviation; Strain Rate Dependence of Stress and Tangent Modulus represents the percent increase for each quantity relative to its 0.5 mm/min value.

Test Rate (mm/min) [Strain Rate]	Treatment	n	Stress (kPa)	Tangent Modulus (MPa)	Strain Rate Dependence	
					Stress	Tangent Modulus
0.5	Control	22	196.59 ± 115.47 ^(a)^	11.88 ± 2.84 ^(g)^		
[6.4 × 10^−4^/s]	Genipin	22	329.06 ± 207.43 ^(b)^	15.62 ± 4.91 ^(h)^		
			*p* ^(a,b)^ = 0.002	*p* ^(g,h)^ = 2.9 × 10^−4^		
10	Control	26	330.95 ± 170.09 ^(c)^	12.50 ± 6.39 ^(i)^	68.3%, *p* ^(a,c)^ = 0.001	5.2%, *p* ^(g,i)^ > 0.05
[0.0128/s]	Genipin	26	401.62 ± 211.08 ^(d)^	17.58 ± 6.30 ^(j)^	22.1%, *p* ^(b,d)^ > 0.05	12.5%, *p* ^(h,j)^ > 0.05
			*p* ^(c,d)^ > 0.05	*p* ^(i,j)^ = 6.8 × 10^−5^		
100	Control	21	484.48 ± 136.62 ^(e)^	17.63 ± 4.46 ^(k)^	146.4%, *p* ^(a,e)^ = 1.8 × 10^−9^	48.4%, *p* ^(g,k)^ = 4.4 × 10^−6^
[0.128/s]	Genipin	21	513.53 ± 254.29 ^(f)^	19.32 ± 5.33 ^(l)^	56.1%, *p* ^(b,f)^ = 0.006	23.7%, *p* ^(h,l)^ = 0.011
			*p* ^(e,f)^ > 0.05	*p* ^(k,l)^ > 0.05		

**Table 2:** Relaxation stress divided by input strain values at 10, 150, and 275 s after the start of relaxation (mean ± standard deviation).

	0.5 mm/min Input Case (n = 13)		100 mm/min Input Case (n = 14)	
Time (s)	Control (MPa)	Genipin (MPa)	Control (MPa)	Genipin (MPa)
10	5.01 ± 1.28	5.89 ± 1.46	3.05 ± 0.71	3.95 ± 0.99
	(*p* = 0.003)		(*p* = 0.005)	
150	3.61 ± 0.85	4.36 ± 1.03	1.76 ± 0.46	2.40 ± 0.59
	(*p* = 0.002)		(*p* = 0.004)	
275	3.25 ± 0.75	3.97 ± 0.91	1.54 ± 0.41	2.11 ± 0.52
	(*p* = 0.002)		(*p* = 0.004)	

## Data Availability

The original contributions presented in the study are included in the article. The data supporting the conclusions of this study are available from the corresponding author upon reasonable request.
